# The Novel Competing Endogenous Long Noncoding RNA SM2 Regulates Gonadotropin Secretion in the Hu Sheep Anterior Pituitary by Targeting the Oar-miR-16b/TGF-β/SMAD2 Signaling Pathway

**DOI:** 10.3390/cells11060985

**Published:** 2022-03-14

**Authors:** Zhen Wan, Hua Yang, Peiyong Chen, Zhibo Wang, Yu Cai, Xiaolei Yao, Feng Wang, Yanli Zhang

**Affiliations:** Jiangsu Livestock Embryo Engineering Laboratory, Nanjing Agricultural University, Nanjing 210095, China; 2019105038@njau.edu.cn (Z.W.); 2018205011@njau.edu.cn (H.Y.); 2020105038@stu.njau.edu.cn (P.C.); 2020205010@stu.njau.edu.cn (Z.W.); 2018105027@njau.edu.cn (Y.C.); yaoxiaolei@njau.edu.cn (X.Y.); caeet@njau.edu.cn (F.W.)

**Keywords:** pituitary cells, lncRNA SM2, TGF-β/SMAD2, oar-miR-16b, gonadotropin

## Abstract

Pituitary gonadotropins play a pivotal role in reproduction. Long noncoding RNAs (lncRNAs) have been identified as important regulators in the hypothalamic–pituitary–ovarian (HPO) axis associated with reproduction. However, the contributions of lncRNAs to pituitary gonadotropin secretion remain largely unknown. Therefore, this work was performed to uncover the functional mechanisms of the novel lncRNA TCONS_00083279 (lncRNA SM2) and its potential targeting pathway oar-miR-16b/TGF-beta/SMAD2, which is associated with gonadotropin secretion in sheep pituitary cells. In the present study, the lncRNA SM2 showed high expression levels in the sheep pituitary gland, and it was located in both the nucleus and the cytoplasm of pituitary cells. lncRNA SM2 knockdown inhibited pituitary cell proliferation and FSH and LH secretion. The function of the lncRNA SM2 was sponged by oar-miR-16b, and this regulated the growth and gonadotropin secretion of pituitary cells by modulating SMAD2, as shown by the dual-luciferase reporter assay. FSH and LH levels were both upregulated by SMAD2 overexpression. Moreover, the levels of the lncRNA SM2, SMAD2 and TGFR1, as well as FSH and LH, in sheep pituitary cells increased significantly under gonadotropin-releasing hormone (GnRH) stimulation (*p* < 0.05). This work illustrates that the lncRNA SM2 regulates gonadotropin secretion in the Hu sheep anterior pituitary by targeting the oar-miR-16b/TGF-β/SMAD2 signaling pathway, providing a valuable resource for understanding the molecular mechanisms underlying sheep reproduction.

## 1. Introduction

The pituitary is composed of the neurohypophysis and the adenohypophysis in mammals, the latter of which constitutes the functional link between the nervous system and endocrine system [1]. The adenohypophysis is a key regulator of the hypothalamic–pituitary–gonadal (HPG) axis [2] and is capable of regulating a variety of functions, including stress [3], growth [4] and reproduction [5]. The adenohypophysis can secrete FSH and LH, which are key hormones that regulate reproductive activity. In males, FSH promotes the synthesis of androgen-binding protein in Sertoli cells and thereby affects spermatogenesis, whereas LH can stimulate androgen secretion in Leydig cells [6]. In females, FSH promotes follicle development, maturation and estrogen secretion. Meanwhile, the mid-cycle LH surge can trigger ovulation [7]. The biosynthesis and secretion of gonadotropins are not only regulated by the GnRH and gonadal hormones [8] but also influenced by some noncoding RNAs in pituitary cells. However, the related molecular regulation mechanism is less explained.

lncRNAs, a class of noncoding RNAs with a length greater than 200 nucleotides, function by binding to DNA/RNA or proteins. Studies have shown that lncRNAs regulate the dosage compensation effect [9], epigenetic modification [10], the cell cycle [11] and cell differentiation [12,13]. Specifically, lncRNAs can participate in the molecular regulatory mechanisms governing hormone secretion [14,15], sperm motility [16,17], follicular development [18] and the differentiation of stem cells [19]. Recently, lncRNAs have been reported to act as competing endogenous RNAs (ceRNAs) for miRNAs to regulate target genes’ expression [20]. For example, the lncRNA PVT1 can act as a ceRNA to regulate miR-519d-3p [21]. The function of the lncRNA FDNCR was sponged by miR-543-3p, and this promoted granulosa cells’ apoptosis by modulating the DCN/TGF-β signaling pathway [22]. Although accumulating research has been reported to decipher the functions of lncRNAs [23], it is a major scientific challenge to clarify the detailed molecular mechanisms of how lncRNAs function [24]. lncRNAs have been confirmed to participate in pituitary functions. For example, lncRNA-m433s1 regulates FSH secretion in male rat pituitary cells [25]. The lncRNA MIR205HG regulates growth hormone and prolactin secretion [26]. AFAP1-AS1 regulates cell proliferation by interacting with miR-103a-3p [27]. Additionally, lncRNAs have been identified to associate with pituitary adenomas, such as RPSAP52 [28]. The above results indicate that lncRNAs might play important roles in pituitary development and gonadotropin secretion. However, the expression levels, function and regulatory mechanisms of lncRNAs in pituitary cells need further investigation.

Analyzing the regulation mechanism on the reproductive traits of mutton sheep is an important basis for improving the production efficiency. Hu sheep, an indigenous Chinese breed with high prolificacy, is an ideal object to investigate the mechanism of mutton sheep multiple births. Follicular development and ovulation are mainly controlled by the HPO axis. One study has shown that the LH pulse size secreted by the pituitary gland directly affects the ovulation number of Hu sheep [29]. Additionally, the mean concentrations of FSH and LH in sheep with high fecundity were significantly higher than those in sheep with low fecundity [29,30]. Therefore, it is meaningful to explore the potential mechanism of pituitary gonadotropin secretion to reveal the mechanism of multiple births in Hu sheep. At the same time, an increasing amount of research has proved the TGF-β/SMAD2 signaling pathway can be involved in the reproductive regulation of female animals. Studies have shown that SMAD3 regulates the FSH secretion of pituitary gonadotropin cells [31]. FSH and LH could regulate natriuretic peptide type C levels in mural granulosa cells via TGF-β pathways and then control the process of oocyte meiosis [32]. In our previous study, we identified a differential candidate lncRNA (lncRNA SM2) and its targeted gene *SMAD2* in Hu sheep pituitary glands with high and low prolificacy. However, how the lncRNA SM2 is transcriptionally regulated and whether it is involved in gonadotropin secretion are still unclear. We hypothesized that the lncRNA SM2 could act as a ceRNA to regulate gonadotropin secretion via the TGFβ/SMAD2 signaling pathway in Hu sheep pituitary cells. Our present study found lncRNA SM2 knockdown reduced the secretion of gonadotropins, inhibited cell proliferation and promoted cell apoptosis in vitro. Mechanistically, we identified the cytoplasmic localization of the lncRNA SM2 in Hu sheep pituitary cells and demonstrate that the lncRNA SM2 acted as a ceRNA to regulate gonadotropin secretion in pituitary cells via the TGF-β/SMAD2 pathway by acting as a molecular sponge to oar-miR-16b. Interestingly, oar-miR-16b could promote the apoptosis of pituitary cells but inhibit pituitary cell proliferation. Furthermore, SMAD2 had a close association with gonadotropin secretion in pituitary cells. Taken together, this study focused on the regulatory mechanism between the lncRNA SM2 and the TGF-β/SMAD2 signaling pathway, thereby identifying a novel molecular pathway of FSH and LH secretion in pituitary cells.

## 2. Materials and Methods

### 2.1. Animals and Sample Collection

Female Hu sheep with similar breeding conditions were fed at the Taizhou Sheep Farm (Jiangsu, China), with free access to food and water. Fifteen female Hu sheep with a similar body weight were selected according to their female parents. They were divided into five groups according to the different stages (5D: fifth day after birth; 3M: before puberty; 6M: before sexual maturity; 9M: after sexual maturity; and 2Y: body mature), and each group contained three Hu sheep. Pituitary samples were collected and stored in liquid nitrogen for total RNA extraction after slaughtering. Various tissues of the 2-year-old Hu sheep were collected and used for tissue expression analysis.

### 2.2. Cell Culture and Treatments

Five female pituitary glands from three-month-old Hu sheep were collected for isolating primary pituitary cells from a local abattoir (Taicang, Jiangsu, China; 121°10′ E, 31°45′ N). Isolation of primary pituitary cells was conducted using collagenase IV (1 mg/mL, 15 min) and Trypsin (0.25%, 15 min) in the lab. The pituitary cells were cultured in DMED/DF12 (Gibco Life Technologies, Grand Island, NY, USA) containing 10% FBS (Gibco Life Technologies, Grand Island, NY, USA) and 100 U/mL penicillin. Additionally, 293T cells were grown in DMEM (Gibco Life Technologies, Grand Island, NY, USA) containing 10% FBS (Gibco Life Technologies, Grand Island, NY, USA) and 100 U/mL penicillin at 37 °C in a 5% CO_2_ atmosphere. 

### 2.3. Vector Construction

The coding DNA sequence region of SMAD2 (GenBank: XM_015103722.3) was cloned into a pEX overexpression vector. To identify the siRNA of the lncRNA SM2 with the highest interference efficiency, three siRNAs were designed, which were named siRNA1-LncRNA SM2-2592, siRNA2-LncRNA SM2-3415 and siRNA3-LncRNA SM2-3794. The siRNAs of the lncRNA TCONS-00083279, SMAD2, negative control (NC) and the oar-miR-16b mimics and inhibitor were synthesized by the GenePharma company (Shanghai, China). The sequences of siRNAs are listed in Table 1.

### 2.4. Cell Transfection and Treatment

Pituitary cells were seeded into 6-well plates for siRNA transfection. When pituitary cells reached 60–70% confluence, the siRNA/pEX-SMAD2 was mixed with the Lipofectamine 3000 reagent (Invitrogen, Waltham, CA, USA) and added to 6-well plates seeded with pituitary cells, according to the manufacturer’s protocol. Meanwhile, GnRH powder (Ningbo Second Hormone Factory, Ningbo, China) was dissolved to 1000 nM with DMEM/F12 as a concentrated stock solution and then gradually diluted with DMEM/F12 to the corresponding concentration (100 nM, 10 nM, 1 nM), according to the reference of the relevant studies [33,34,35]. When pituitary cells reached 60–70% confluence in 6-well plates, they were treated with different concentrations of GnRH in a medium for 24 or 48 h, with a total of three replicates for each concentrated treatment. The cells were harvested 24 h after transfection for RNA extraction and 48 h after transfection for protein extraction.

### 2.5. lncRNA Coding Ability Prediction and miRNA Prediction

CPC (http://cpc.cbi.pku.edu.cn/ (accessed on 28 April 2021)) software was used to identify the coding ability of the lncRNA SM2.

SMAD2 3′ UTR sequences were obtained from NCBI (XM_027960885). Interactions involving miRNA–mRNA and lncRNA SM2–miRNA were predicted using miRanda (https://www.miranda-ng.org/en/ (accessed on 4 April 2021)) (score > 140; energy threshold ≤−20) and RNAhybrid (https://bibiserv.cebitec.uni-bielefeld.de/rnahybrid (accessed on 4 April 2021)) (energy threshold ≤ −20).

### 2.6. Quantitative Real-Time Polymerase Chain Reaction (qPCR)

TRIzol Reagent (Invitrogen, Waltham, CA, USA) was used to extract total RNA from the Hu sheep pituitary. Then, reverse transcription was performed according to the PrimerScript RT reagent kit instructions (Vazyme, Nanjing, China). Additionally, RT-qPCR was performed on the StepOnePlus Real-Time PCR system (Life Technologies, New York, NY, USA), as previously described [36]. The differential expression changes of genes were quantified by the 2^−ΔΔCt^ method. U6 [37] (for miRNA) and β-actin [38] (ACTB) were used as controls. Each experiment was repeated at least three times. The primers used for qPCR are listed in Table 2 and Table 3.

### 2.7. EDU Assay

Cell proliferation was detected using the kFluor555 Click-iT EdU Assay Kit (KGA337-500, KeyGEN BioTECH, Jiangsu, China). The specific steps were as follows: Pituitary cells were seeded into 24-well plates plated with sterile cell slides in advance, after transfection treatment, and then the Edu kit was used for detection. The original 10 μM Edu working solution was diluted to 50 nM with complete medium and treated with pituitary cells for 2 h. According to the kit instructions, the Click-iT reaction mixture was prepared, with 0.5 mL added to each well, shaken gently and incubated at room temperature for 30 min to avoid light. After incubation, the reaction solution was removed and washed 3 times with DPBS containing 3% BSA. An amount of 0.5 mL of Hoechst staining solution with a final concentration of 5 μg/mL was added to each well, incubated at room temperature for 20 min to avoid light and washed 3 times with DPBS. Finally, the anti-fluorescence quencher was added and examined under a laser scanning confocal microscope (Carl Zeiss, Oberkochen, Germany). Data analysis was performed with Image J software (ImageJ (nih.gov), accessed on 15 April 2021).

### 2.8. Flow Cytometry Analysis

Cell apoptosis was detected using the PE Annexin V Apoptosis Detection Kit I (No. 559763, BD Biosciences Pharmingen, San Diego, CA, USA), according to the manufacturer’s instructions. After treatment, cells were digested with 0.05% Trypsin without EDTA for 5 min and harvested for flow cytometry. Then, the cells were washed 3 times with DPBS. After centrifugation at 1500 rpm for 5 min, the supernatant was discarded, and the cells were stained with 7-Amino-Actinomycin and Annexin V phycoerythrin for 15 min. Finally, detection of apoptosis was carried out by flow cytometry (Beckman Coulter, Brea, CA, USA). The apoptosis data were analyzed using FlowJo 7.6.

### 2.9. Immunofluorescence (IF)

Pituitary cells were seeded in 24-well plates containing slides, and when the cells reached 60–70% confluence, they were fixed in 4% paraformaldehyde for 1 h. Additionally, the cells were permeabilized with 0.2% Triton X-100 for 15 min; washed 3 times with 1× PBS, 5 min/time; and then blocked in 5% bovine serum albumin (BSA) for 2 h at room temperature and incubated with Anti-LHβ (1:100, AFFinity, Sterling, VA, USA) and Anti-FSHβ antibody (1:100, Abcam, Cambridge, UK) at 4 °C overnight. Next, the samples were washed with 1× PBS and incubated with Alexa fluor goat anti-rabbit secondary antibody to avoid light (1:1000, Proteintech, Wuhan, China) for 2 h. Then, the nucleus was incubated with 4′,6-diamidino-2-phenylindole (DAPI) for 10 min and washed 3 times with 1× PBS. Finally, slides were examined using a laser scanning confocal microscope (Carl Zeiss, Oberkochen, Germany).

### 2.10. Western Blot

Protein was harvested from the treated pituitary cells with RIPA buffer (Thermo Pierce, Rockford, IL, USA), and the supernatant obtained by high-speed centrifugation was used to determine the protein concentration with the enhanced BCA protein assay kit (Beyotime, Wuhan, China). Next, the supernatant was mixed with Nupage LDS sample buffer (5×) and Nupage sample reduction agent (10×) (Thermo Pierce, Rockford, IL, USA) in a 100 uL system and boiled at 70 °C for 10 min to denature. A 12% SDS-PAGE gel for electrophoresis was used for separating 20 μg proteins per lane, which were subsequently transferred to a polyvinylidene fluoride membrane (Millipore, MA, USA). The membranes were blocked with 5% fat-free milk powder for 1.5 h and then exposed to the primary antibody (diluted according to Table 4) overnight at 4 °C. The internal control was β-actin. Then, HRP-goat anti-rabbit IgG was incubated for 1.5 h. Finally, immunoblotting was conducted with Image Quant LAS 400 (Fiji film, Tokyo, Japan).

### 2.11. FISH Analysis

The lncRNA SM2 FISH probe mix (Cy3-labeled) for RNA FISH analysis was used to evaluate the localization of the lncRNA SM2 in pituitary cells with a FISH kit (GenePharma, F12201/50, Shanghai, China). The nucleus was stained with DAPI. 18S was used as a positive control for the cytoplasm. The specific experimental steps followed the manufacturer’s instructions.

### 2.12. ELISA Assay

After 48 h of transfection, the cell supernatant was collected, and gonadotropins were determined using the ELISA assay (Amresco, Shanghai, China) (FSH: DRE-S9373, LH: DRE-S9371), according to the manufacturer’s protocol. The sensitivity of this assay is 0.1 mIU/mL. The values of the inter- and intra-variation coefficient for the kit were <10%.

### 2.13. Dual-Luciferase Reporter Assays

We constructed wild-type plasmids lncRNA SM2-WT and SMAD2-WT, as well as mutant-type plasmids lncRNA SM2-MUT and SMAD2-MUT. Pituitary cells seeded into 24-well plates were subject to transfection with 100 nM oar-miR-16b mimics or a negative control and 0.5 ng mutant-type or wild-type plasmid with Lipofectamine 3000. After 48 h of transfection, pituitary cells were collected, and luciferase activity was determined using the Dual-Luciferase reporter assay system (Vazyme, Nanjing, China), according to the manufacturer’s protocol.

### 2.14. Data Analysis

All experiments were repeated at least three times. Statistical analysis of the data was performed using SPSS software (SPSS Inc., Chicago, IL, USA). The significance of differences between samples was assessed by a t-test for two-group comparisons, and by one-way ANOVA for three or multiple groups. The data were expressed as the mean ± standard error (SEM). Values of *p* < 0.05 (*) and *p* < 0.01 (**) indicate significant and extremely significant differences, respectively.

## 3. Results

### 3.1. lncRNA SM2 Was Highly Expressed in Pituitary Gland

**Figure 1:**
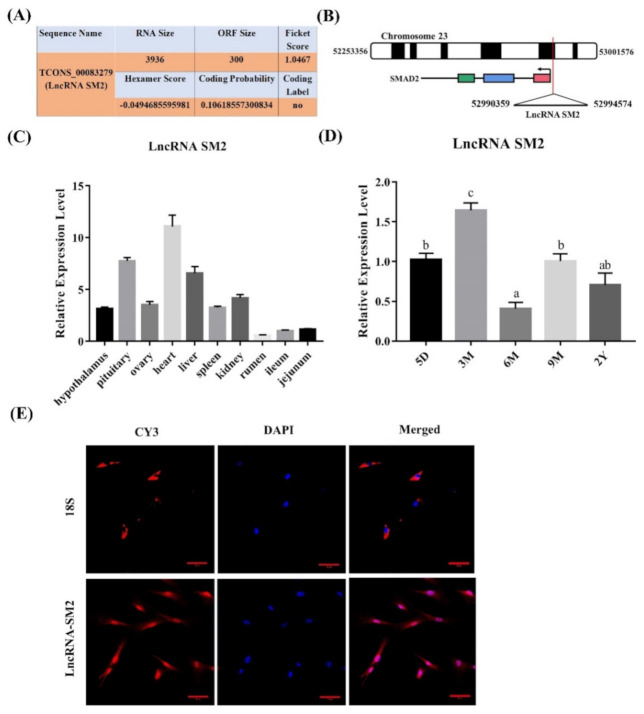
Characterization of a candidate transcript in Hu sheep. (**A**) The coding ability of lncRNA SM2 was predicted using Coding Potential Assessment Tool (CPAT). (**B**) Chromosomal localization of lncRNA SM2. (**C**,**D**) Expression of lncRNA SM2 in each group. (**E**) lncRNA SM2 localization in Hu sheep pituitary cells; scale bar, 50 μm. All data are shown as the mean ± SEM of at least three replicates. Different letters indicate significant differences between groups, *p* < 0.05.

To analyze the functions of the novel lncRNA SM2 identified in our previous study based on Hu sheep pituitary transcriptomic results associated with prolificacy [39], we chose the lncRNA SM2 as the candidate lncRNA. The novel lncRNA SM2 is an intergenic lncRNA located on chromosome 23 (Figure 1B). We also used CPC software to identify the coding ability of the lncRNA SM2, and the results show that it did not encode proteins (Figure 1A). Furthermore, tissue expression analysis in various female Hu sheep found that the lncRNA SM2 was expressed in most organs, including the heart, liver, spleen, kidney, rumen, jejunum, ileum, hypothalamus, ovary and pituitary gland, and it had the highest expression in the heart and pituitary gland (Figure 1C). Furthermore, the lncRNA SM2 had the highest expression in the pituitary gland of three-month-old Hu sheep (Figure 1D). The FISH assay result shows that the lncRNA SM2 was located in both the nucleus and the cytoplasm (Figure 1E).

### 3.2. lncRNA SM2 Knockdown Downregulated the Secretion of Gonadotropins in Pituitary Cells

**Figure 2:**
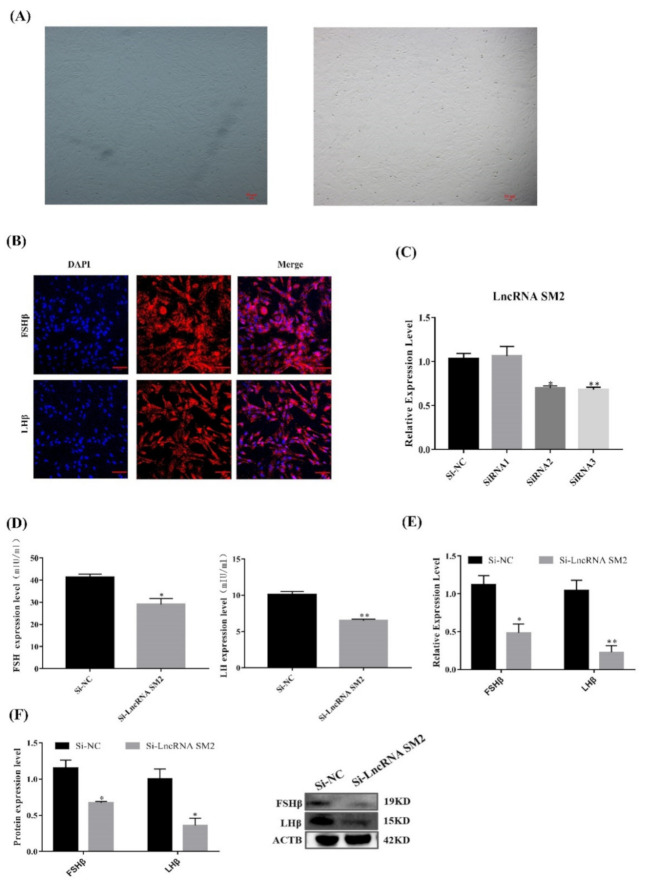
LncSM2 knockdown inhibited the secretion of gonadotropins in pituitary cells. (**A**) Morphology of pituitary cells under a microscope. The left picture shows F1 generation pituitary cells, and the right picture shows F2 generation pituitary cells. (**B**) Identification of sheep pituitary cells by FSHβ and LHβ marker gene immunofluorescence staining. Scale bar, 100 μm. (**C**) mRNA expression of lncRNA SM2 in sheep pituitary cells treated with si-lncRNA SM2-2592, si-lncRNA SM2-3415 and si-lncRNA SM2-3794 was determined by qPCR. (**D**) FSH and LH secretion was detected by ELISA. (**E**,**F**) mRNA and protein expression of FSHβ and LHβ in pituitary cells in each group. All data are shown as the mean ± SEM of at least three replicates (* *p* < 0.05; ** *p* < 0.01).

Hu sheep pituitary cells were isolated and showed a fusiform shape after being attached to the cell culture dish under the microscope (Figure 2A). Immunofluorescence analysis showed that the marker genes *FSHβ* and *LHβ* were expressed in most cells (Figure 2B).

To identify the function of the lncRNA SM2 in pituitary gonadotropin secretion, lncRNA SM2 siRNAs were transfected into pituitary cells to inhibit lncRNA SM2 expression (Figure 2C). The qPCR result shows that siRNA3 (siRNA-LncSM2-3794) had the highest interference efficiency. Therefore, siRNA-LncSM2-3794 was used for subsequent experiments. ELISA showed that gonadotropin levels were significantly decreased (*p* < 0.05) and accompanied by a decreased (*p* < 0.05) expression of FSHβ and LHβ (Figure 2D,F) in lncRNA SM2 knockdown pituitary cells compared to the NC. Taken together, silencing the lncRNA SM2 reduced gonadotropin secretion in pituitary cells.

### 3.3. lncRNA SM2 Knockdown Compromised Pituitary Cell Proliferation and Viability

**Figure 3:**
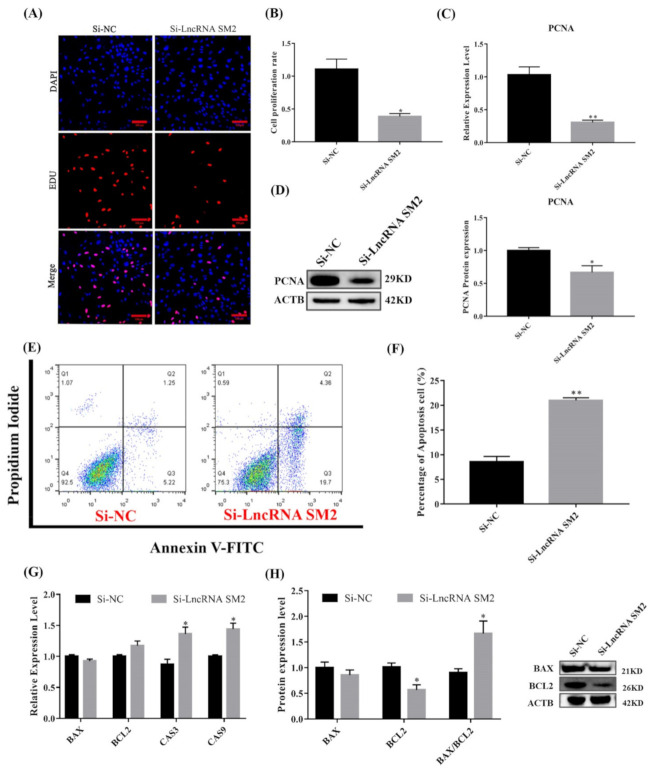
Effect of lncRNA SM2 on proliferation and apoptosis in pituitary cells. (**A**,**B**) Pituitary cells’ proliferation was assayed by EDU. Scale bars, 100 μm. (**C**,**D**) mRNA and protein expression of PCNA in pituitary cells in each group. (**E**,**F**) Pituitary cell apoptosis was detected by flow cytometry. (**G**,**H**) mRNA and protein expression of cell apoptosis-related genes in pituitary cells. All data are shown as the mean ± SEM of at least three replicates (* *p* < 0.05; ** *p* < 0.01).

As shown in Figure 3C,D, cell proliferation marker PCNA mRNA and protein expression were significantly reduced (*p* < 0.05). The Edu results show that the cell proliferation rate was significantly decreased (*p* < 0.05) in lncRNA SM2 knockdown pituitary cells compared to NC pituitary cells (Figure 3A,B). Meanwhile, the expression levels of *Caspase3* and *Caspase9* were increased (*p* < 0.05) in lncRNA SM2 knockdown sheep pituitary cells (Figure 3G). BCL2 protein levels were decreased by Western blot (*p* < 0.05) after the lncRNA SM2 was silenced. Additionally, the ratio of BAX/BCL2 was significantly increased (*p* < 0.05) (Figure 3H). The percentage of apoptotic cells was significantly higher (*p* < 0.01) in the lncRNA SM2 knockdown than in the NC group by flow cytometry (Figure 3E,F). Taken together, these data indicate that the lncRNA SM2 is involved in the regulation of proliferation and the possible prevention of apoptosis in Hu sheep pituitary cells.

### 3.4. lncRNA SM2 Regulates SMAD2 Via oar-miR-16b

**Figure 4:**
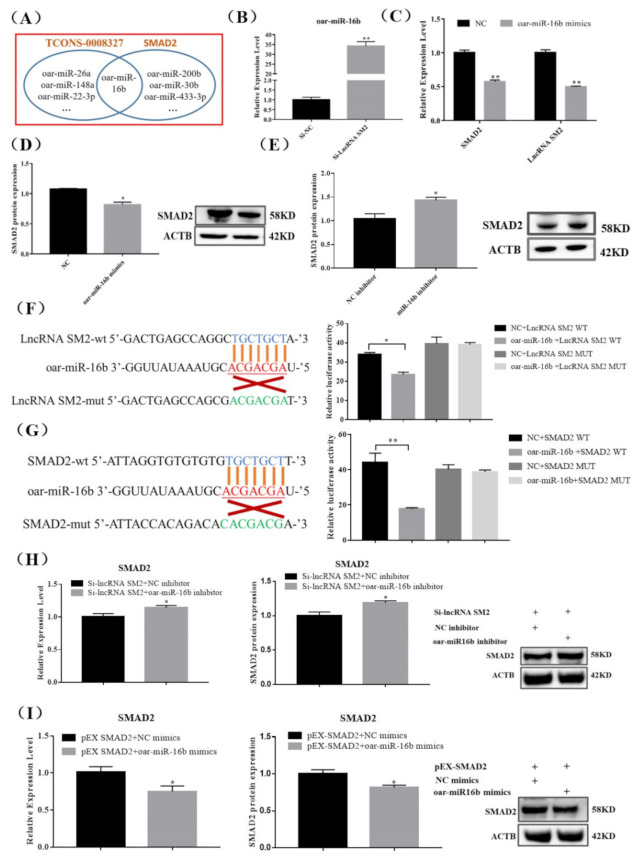
lncRNA SM2 acts as a ceRNA and sponge to oar-miR-16b in the pituitary cells of Hu sheep. (**A**) RNAhybrid and miRanda predicted miRNA targets lncRNA SM2 and SMAD2. (**B**,**C**) Expression of oar-miR-16b, lncRNA SM2 and *SMAD2* in each group by RT-qPCR. (**D**,**E**) SMAD2 protein level was decreased and increased in pituitary cells transfected with oar-miR-16b mimics and inhibitor by Western blot. (**F**,**G**) Schematic depicting the interactions of oar-miR-16b with wild-type lncRNA SM2 or SMAD2 and mutant lncRNA SM2 or SMAD2. The targeting relationship between lncRNA SM2 or SMAD2 and oar-miR-16b was detected by dual-luciferase reporter gene assay. (**H**,**I**) Expression of SMAD2 in each group. All data are shown as the mean ± SEM of at least three replicates (* *p* < 0.05; ** *p* < 0.01).

lncRNAs regulate gene expression transcriptionally or post-transcriptionally. To detect the underlying lncRNA SM2 and SMAD2 association, we predicted the potential binding sites between the lncRNA SM2 and SMAD2. The results show that oar-miR-16b could bind to the lncRNA SM2 and SMAD2 3’-untranslated region (Figure 4A), suggesting that the lncRNA SM2 could act as a ceRNA for oar-miR-16b to mediate SMAD2 expression in pituitary cells. Then, we investigated whether the oar-miR-16b expression level significantly increased (*p* < 0.01) upon lncRNA SM2 silencing in sheep pituitary cells (Figure 4B). Moreover, lncRNA SM2 and *SMAD2* mRNA expression levels decreased (*p* < 0.01) after treatment with oar-miR-16b mimics (Figure 4C). Additionally, the SMAD2 protein expression level was significantly decreased or increased in pituitary cells transfected with the oar-miR-16b mimics or inhibitor (*p* < 0.05) (Figure 4D,E).

Dual-luciferase reporter constructs containing the miRNA response element wild-type (WT) and mutant (MUT) plasmids were co-transfected with oar-miR-16b mimics into pituitary cells. A dual-luciferase reporter assay showed that oar-miR-16b decreased the luciferase activity of the lncRNA SM2-WT construct but not the lncRNA SM2-MUT construct (Figure 4F). In addition, oar-miR-16b also decreased the luciferase activity of the SMAD2-WT construct but not the SMAD2-MUT construct (Figure 4G), indicating a direct relationship between oar-miR-16b and the lncRNA SM2 or SMAD2. Moreover, the oar-miR-16b inhibitor rescued siRNA-lncRNA SM2-reduced SMAD2 expression, and oar-miR-16b mimics inhibited pEX-SMAD2-induced SMAD2 expression (Figure 4H,I). Overall, these data indicate that the lncRNA SM2 regulated SMAD2 by sponging oar-miR-16b.

### 3.5. Effect of Oar-miR-16b Level on Pituitary Cell Proliferation and Apoptosis

**Figure 5:**
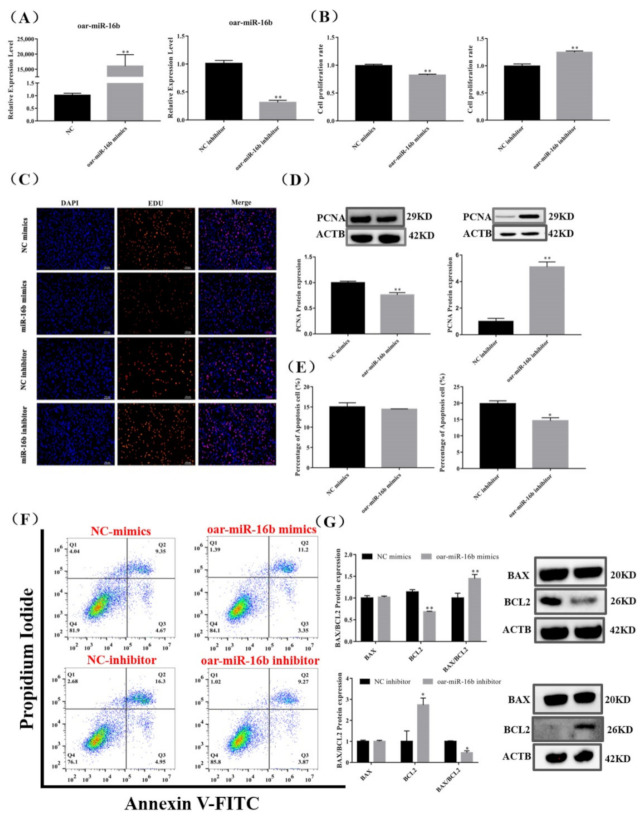
Effect of oar-miR-16b level on pituitary cell proliferation and apoptosis. (**A**) Expression of oar-miR-16b in each group by RT-qPCR. (**B**,**C**) Pituitary cell proliferation was detected by EDU. Scale bars, 100 μm. (**D**) Protein expression of PCNA in pituitary cells. (**E**,**F**) Pituitary cell apoptosis was detected by flow cytometry. (**G**) Protein expression of cell apoptosis-related genes in pituitary cells. All data are shown as the mean ± SEM of at least three replicates (* *p* < 0.05; ** *p* < 0.01).

To examine the function of oar-miR-16b on pituitary cell proliferation and apoptosis, we transfected pituitary cells with mimics and inhibitor of oar-miR-16b. The expression of oar-miR-16b significantly increased and decreased in pituitary cells transfected with the oar-miR-16b mimics and inhibitor (*p* < 0.01) (Figure 5A). Obviously, the oar-miR-16b mimics significantly decreased the proliferation rate of pituitary cells and the protein expression of PCNA (*p* < 0.01) (Figure 5B–D). On the contrary, the oar-miR-16b inhibitor significantly upregulated the proliferation rate of pituitary cells and the protein expression of PCNA (*p* < 0.01) (Figure 5B–D).

Meanwhile, we examined the protein expression of apoptosis markers. The oar-miR-16b mimics significantly inhibited the protein expression of BCL2 and significantly increased the ratio of BAX/BCL2 (*p* < 0.01), but the percentage of apoptotic cells did not change significantly (*p* < 0.05) (Figure 5E–G). Conversely, the oar-miR-16b inhibitor significantly promoted the protein expression of BCL2 and significantly reduced the ratio of BAX/BCL2, and the percentage of apoptotic cells was also significantly reduced (*p* < 0.05) (Figure 5E–G). Taken together, our results indicate that oar-miR-16b promoted the apoptosis of pituitary cells but inhibited pituitary cell proliferation.

### 3.6. SMAD2 Overexpression Upregulates the Secretion of Gonadotropins in Pituitary Cells

**Figure 6:**
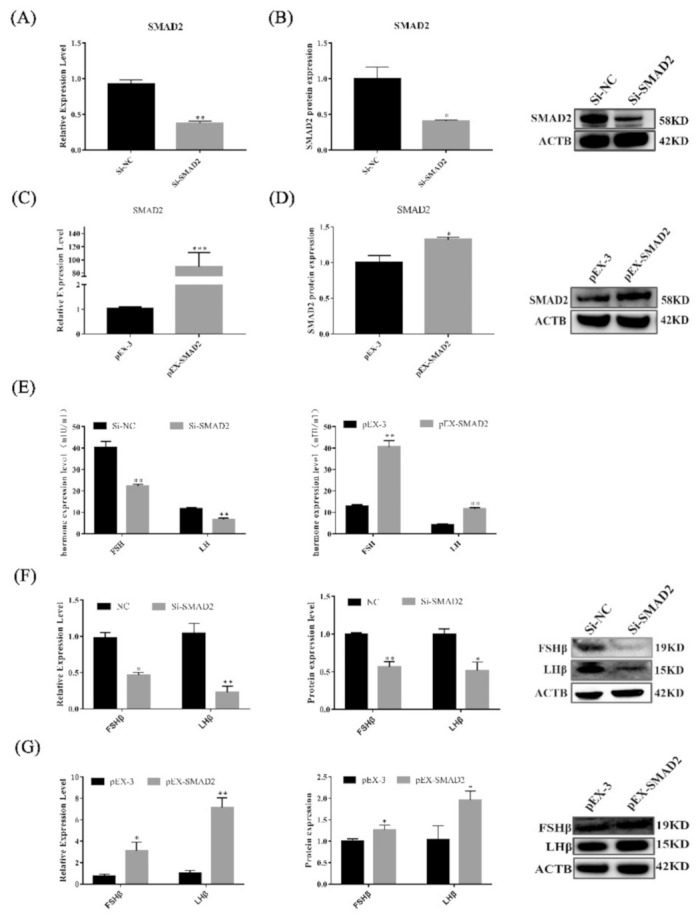
SMAD2 overexpression and knockdown affected the secretion of gonadotropins in pituitary cells. (**A**–**D**) mRNA and protein expression of SMAD2 in pituitary cells in each group. (**E**) FSH and LH secretion was detected by ELISA. (**F**,**G**) mRNA and protein expression of FSHβ and LHβ in pituitary cells in each group. All data are shown as the mean ± SEM of at least three replicates (* *p* < 0.05; ** *p* < 0.01).

To test whether SMAD2 regulates the secretion of gonadotropins in vitro, transfection was used to generate pituitary cells with SMAD2 overexpression or SMAD2 knockdown (Figure 6A–D). SMAD2 overexpression not only elevated the secretion of gonadotropins (*p* < 0.01) but also upregulated the expression levels of the LHβ and FSHβ genes and proteins (*p* < 0.05) (Figure 6E,G). However, knockdown of SMAD2 suppressed the secretion of gonadotropins in pituitary cells(Figure 6E,F). These results indicate that SMAD2 is important to maintain the secretion of gonadotropins in Hu sheep pituitary cells.

### 3.7. SMAD2 Overexpression Upregulated Pituitary Cell Proliferation and Viability

**Figure 7:**
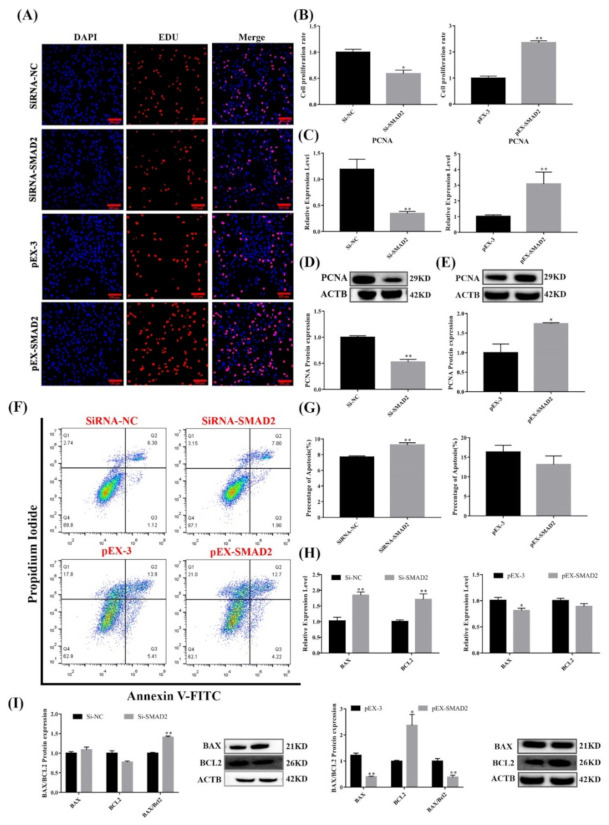
Effect of SMAD2 on proliferation and apoptosis in pituitary cells. (**A**,**B**) Pituitary cell proliferation was detected using EDU. Scale bars, 100μm. (**C**–**E**) mRNA and protein expression of PCNA in pituitary cells in each group. (**F**,**G**) Pituitary cell apoptosis was assayed by flow cytometry. (**H**,**I**) mRNA and protein expression of cell apoptosis-related genes in pituitary cells in each group. All data are shown as the mean ± SEM of at least three replicates (* *p* < 0.05; ** *p* < 0.01).

As shown in Figure 7, SMAD2 overexpression significantly promoted pituitary cell proliferation. Edu incorporation (Figure 7A,B) and PCNA mRNA and protein (Figure 7C,E) expression levels were significantly increased (*p* < 0.05). Furthermore, Western blot analysis revealed that BCL2 protein levels increased (*p* < 0.05) in sheep pituitary cells after SMAD2 was overexpressed (Figure 7I). Additionally, the ratio of BAX/BCL2 was significantly decreased (*p* < 0.01). Flow cytometry showed that the percentage of apoptotic cells was lower (*p* > 0.05) in lncRNA SM2-overexpressing sheep pituitary cells than in the NC group (Figure 7F,G). More importantly, contrasting results were found with SMAD2 overexpression and knockdown, clearly demonstrating that SMAD2 participated in pituitary cell proliferation and cell apoptosis.

### 3.8. lncRNA SM2 Is Involved in Pituitary Cells by Regulating the TGF-β/SMAD2 Pathway

**Figure 8:**
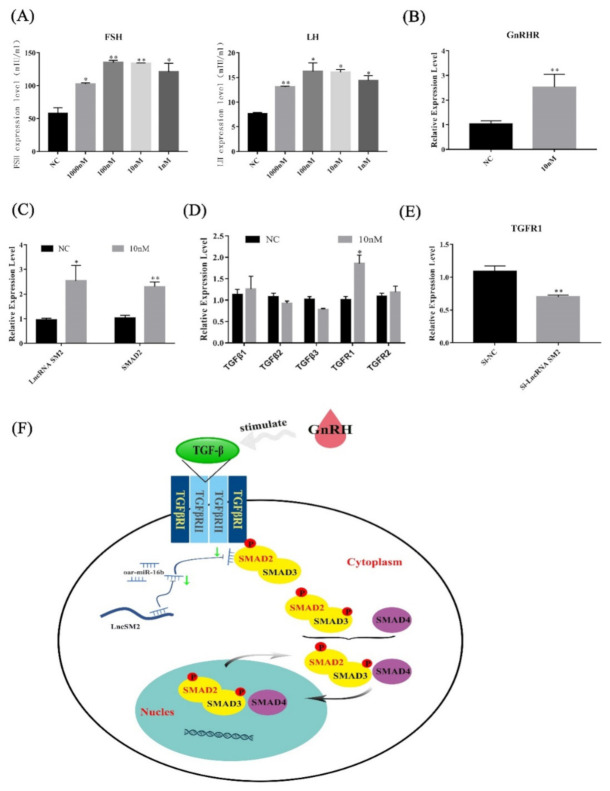
lncRNA SM2 regulated the secretion of gonadotropins by TGFR1/SMAD2 signaling pathways in the pituitary cells of Hu sheep. (**A**) FSH and LH secretion was detected after stimulation with GnRH by ELISA. (**B**–**D**) mRNA expression of *GnRHR*, *lncRNA SM2*, *SMAD2* and *TGF* family in pituitary cells in each group by RT-qPCR. (**E**) mRNA expression of *TGFR1* in pituitary cells in each group by RT-qPCR. (**F**) Proposed model of lncRNA SM2 regulation of pituitary cell state in Hu sheep. All data are shown as the mean ± SEM of at least three replicates (* *p* < 0.05; ** *p* < 0.01).

Luciferase activity analysis revealed that SMAD2 is the target gene of the lncRNA SM2. SMAD2 is an important part of the TGF-β signaling pathway. We further assessed how the lncRNA SM2 acts on pituitary cell gonadotropin secretion via the TGF-β/SMAD pathway. We first cultured pituitary cells under different concentrations of GnRH (Figure 8A), and the ELISA results show that the secretion of gonadotropins in pituitary cells was significantly increased (*p* < 0.05). Then, we selected the 10 nM concentration of GnRH to continue the experiment. qPCR showed that *gonadotropin releasing hormone receptor* (*GnRHR)*, *lncRNA SM2* and *SMAD2* mRNA expression increased significantly (*p* < 0.05) (Figure 8B,C). Meanwhile, we investigated the expression of each subunit in the TGF-β pathway. Additionally, it was found that *TGFR1* mRNA expression increased significantly (*p* < 0.05) after GnRH stimulation (Figure 8D). Conversely, *TGFR1* mRNA expression was decreased significantly (*p* < 0.01) in lncRNA SM2 knockdown sheep pituitary cells (Figure 8E). Taken together, these data show that the lncRNA SM2 regulated the secretion of gonadotropins in Hu sheep pituitary cells via the TGFR1/SMAD2 pathway (Figure 8F).

## 4. Discussion

Several studies have shown that lncRNAs play essential roles in the regulation of reproduction in mammals. Gonadotropins secreted by the adenohypophysis can affect the quality of fecundity. Therefore, in addition to well-characterized lncRNAs, it is worth exploring potential essential lncRNAs that control the mechanisms of gonadotropin secretion. In our previous study, we identified a differentially expressed lncRNA by sequence analysis, and its target gene was *SMAD2*. We found that the lncRNA SM2 was highly expressed in the pituitary gland. Interestingly, we found that the expression levels of the lncRNA SM2 increased significantly in pituitary cells treated with GnRH in our study. Therefore, the biological function of the lncRNA SM2 in the pituitary gland is worth further investigating.

At present, the potential functions and regulatory mechanisms of lncRNAs in FSH and LH secretion are being increasingly studied. Here, we performed loss-of-function assays to determine the role of the lncRNA SM2 in pituitary cells. Interestingly, our results show that lncRNA SM2 knockdown suppressed cell proliferation and gonadotropin secretion but facilitated cell apoptosis. Our results demonstrate that the lncRNA SM2 might regulate the levels of gonadotropin secretion through certain underlying mechanisms.

It is well known that lncRNAs, commonly functioning as ceRNAs, for example, can upregulate a certain mRNA by sponging miRNA [40,41]. The FISH results show that the lncRNA SM2 is mainly located in the cytoplasm, indicating its post-transcriptional regulatory mechanism in pituitary cells. We hypothesized that the lncRNA SM2 regulates SMAD2 by acting as a ceRNA to sponge miRNAs. Similarly, we predicted miRNAs that are co-bound between lncRNAs and their target gene *SMAD2*. Among all candidates, oar-miR-16b has a simultaneous potential targeted binding site with the lncRNA SM2 and SMAD2. Our current study found that oar-miR-16b expression levels significantly increased upon lncRNA SM2 silencing in pituitary cells. The dual-luciferase reporter results reveal that oar-miR-16b is a target of the lncRNA SM2 and SMAD2. Moreover, lncRNA SM2 and SMAD2 levels decreased after treatment with oar-miR-16b mimics. Co-transfection experiments further indicated that the lncRNA SM2 could competitively bind to oar-miR-16b for binding and might alleviate the inhibitory effect of oar-miR-16b on the target gene *SMAD2*, which confirmed our hypothesis. Taken together, our experimental results show that the lncRNA SM2 exerts its effects partly by sponging oar-miR-16b in pituitary cells.

In contrast to lncRNAs, miRNAs are a class of endogenous noncoding RNA transcripts with a length greater than approximately 19–24 nt. In animals, miRNAs mainly bind the 3’-untranslated region (UTR) through incomplete complementarity, which then results in translational repression or degradation of downstream target mRNAs [42]. In general, a single miRNA can target potentially hundreds or even thousands of mRNA transcripts in mammals [43]. It has been reported that miRNAs are abundant in the central nervous system [44]. miRNAs have been shown to regulate FSHβ expression and further affect FSH secretion, such as miR-361-3p [45] and miRNA-7 [46]. In this study, we found that a known miRNA may be involved in the regulation of pituitary function. However, the precise functions of oar-miR-16b in the pituitary cells are still unclear. We demonstrated that inhibition of oar-miR-16b could promote cell proliferation and inhibit cell apoptosis. Additionally, oar-miR-16b is functionally opposite to the target gene *SMAD2* in the pituitary cells. Our data enrich the research on the functional mechanism underlying the regulatory role of miRNA in hormone secretion in the Hu sheep pituitary gland.

The SMAD family of proteins acts as a bridge between TGF-β proteins and transmits their signals from transmembrane receptors to the nucleus. The TGF-β/SMAD2 pathway is the classic pathway for the TGF-β family to transmit signals. Previous mouse model studies have shown that deletion of SMAD4 and FOXL2 in GnRH results in FSH deficiency and female infertility [47], and FSH and LH can enhance the activity of the TGF-β/SMAD signaling pathway [48]. Moreover, SMAD2 downregulation could promote cell apoptosis and inhibit cell proliferation, thereby suppressing testosterone levels in sheep Leydig cells [49]. In our study, we determined that SMAD2 is a potential downstream regulator of oar-miR-16b by dual-luciferase reporter analysis. Therefore, it is important to explore its downstream molecular mechanism. In this study, we found that the expression level of *SMAD2* in Hu sheep pituitary glands of different ages was consistent with that of the lncRNA SM2 (Figure S1). SMAD2 overexpression significantly promoted pituitary cell proliferation and suppressed apoptosis. Additionally, SMAD2 overexpression caused significant increases in FSH and LH levels in pituitary cells. Therefore, SMAD2 could also regulate gonadotropin levels by affecting cell proliferation and apoptosis in pituitary cells, and we confirmed that SMAD2 was the functional target of the lncRNA SM2/oar-miR-16b. To further determine whether the lncRNA SM2 directly regulates gonadotropin secretion, we used GnRH to stimulate pituitary cells. Recent reports indicated that GnRH analogue treatment activated SMAD3 and SMAD4 in human endometrial stromal cells [50]. Our results indicate that GnRH treatment promoted *SMAD2* and *TGFR1* expression in pituitary cells. Moreover, SMAD2 was dispensable for FSHB and GnRHR expression by gonadotropes in mice [51], and this may indicate the species-related differences in the molecular regulation of gonadotrophin secretion. Based on all these data, we conclude that the lncRNA SM2 suppressed pituitary cell survival and gonadotropin secretion via the TGF-β/SMAD2 signaling pathway in Hu sheep.

In conclusion, numerous lncRNAs of different abundance in the Hu sheep pituitary gland were annotated. We further identified the lncRNA SM2 in pituitary cells. Our findings confirm the mechanism whereby the lncRNA SM2 sponges oar-miR-16b in pituitary cells and prevents oar-miR-16b from binding to SMAD2 through the TGF-β pathway to promote pituitary cell hormone secretion in Hu sheep. Therefore, this study identified a candidate lncRNA (lncRNA SM2) involved in Hu sheep fecundity, providing insights into the regulatory mechanisms underlying hormone secretion, which will provide a basis for new therapeutic strategies for reproductive diseases.

## Figures and Tables

**Table 1:** Details of siRNA sequences used for cell transfection.

Items	Primer Sequence (5′-3′)
siRNA-NC	S: 5′-UUCUCCGAACGUGUCACGUTT-3′
	A: 5′-ACGUGACACGUUCGGAGAATT-3′
siRNA-LncSM2-1 (3415)	S: 5′-GCGGCCAGAAUGUAAUUAATT-3′
	A: 5′-UUAAUUACAUUCUGGCCGCTT-3′
siRNA-LncSM2-2 (2592)	S: 5′-GCUGGAGAAUAAAGCAUAUTT-3′
	A: 5′-AUAUGCUUUAUUCUCCAGCTT-3′
siRNA-LncSM2-3 (3794)	S: 5′-CUCCAACACUAGGCAAAUATT-3′
	A: 5′-UAUUUGCCUAGUGUUGGAGTT-3′
siRNA-SMAD2-531	S: 5′-GCCUGAUCUUCACAGUCAUTT-3′
	A: 5′-AUGACUGUGAAGAUCAGGCTT-3′
oar-miR-16b mimics	S: 5′-UAGCAGCACGUAAAUAUUGG-3′
	A: 5′-AAUAUUUACGUGCUGCUAUU-3′
NC mimics	S: 5′-UUCUCCGAACGUGUCACGUTT-3′
	A: 5′-ACGUGACACGUUCGGAGAATT-3′
oar-miR-16b inhibitor	5’-CCAAUAUUUACGUGCUGCUA-3’
NC inhibitor	5’-CAGUACUUUUGUGUAGUACAA-3’

**Table 2:** Primer sequences of genes used for reverse transcription and quantitative real-time PCR.

Gene Name	Forward Primer (5′-3′)	Reverse Primer (5′-3′)	Usage
*ACTB*	CGCAAGTACTCCGTGTGGAT	TAACGCAGCTAACAGTCCGC	RT-qPCR
*LncSM2*	TCTAGAACGCAAGTGCCTGG	GTCTGCAAGGAAAAGGCGAC	RT-qPCR
*SMAD2*	GCAATCTTTGTGCAGAGCCC	TGCTTGTTACCGTCTGCCTT	RT-qPCR
*PCNA*	CCTTGGTGCAGCTAACCCTT	GCCAAGGTGTCCGCATTATC	RT-qPCR
*BAX*	TTGGCTGAGTCGCTGAAGAGC	AACTCCCATGGCCCCCAAAT	RT-qPCR
*BCL2*	CCTTTCGTTTGCTCGTGCTC	ACCTCCTCCGTGATGTGGTAT	RT-qPCR
*FSHB*	GCTATTGCTACACCCGGGAC	AGTGGCTACTGGGTACGTGT	RT-qPCR
*LHB*	CCGCTCCCAGATATCCTCTTC	TTATTGGGAAGGGAGGGGAGG	RT-qPCR
*GnRHR*	GCTGCCTCTTCATCATCCCTCTTC	CCTCAGCCGAGCTTGTGGTATATTG	RT-qPCR
*TGF-β1*	ACCGGCCCTTCCTGCTCCTCAT	GGAGCGCACGATCATGTTGGA	RT-qPCR
*TGF-β2*	CCCCAGAAGACTACCTCG	AGTATTCCTCGTCGCTCC	RT-qPCR
*TGF-β3*	CCACCTTGGACTTCAACC	CGGGTGCTGTTGTAAAGA	RT-qPCR
*TGF-R1*	CTGTCGGAAAGCCGTCATCT	TCCTCTTCACTTGGCACTCG	RT-qPCR
*TGF-R2*	TCGCCGAGGTCTACAAGG	GCGTGGAAGGCAGTGATG	RT-qPCR
*U6*	CTCGCTTCGGCAGCACA	AACGCTTCACGAATTTGCGT	RT-qPCR

**Table 3:** Primer sequences of miRNAs.

Genes	Primer Sequence (5′-3′)	Usage
*oar-miR-16b*	GTCGTATCCAGTGCAGGGTCCGAGGTATTCGCACTGGATACGACCCAATA	RT-qPCR
*oar-miR-16b*	F: GCGCGTAGCAGCACGTAAA	RT-qPCR
	R: AGTGCAGGGTCCGAGGTATT	RT-qPCR

**Table 4:** Details of specific antibody used for Western blot and IF in the experiment.

Antibodies	Cat No.	Source	Dilution of IF	Dilution of WB
SMAD2	12570-1-AP	Proteintech, Wuhan, China	1:100	1:1000
PCNA	AF0239	AFFinity, Sterling, VA, USA		1:1000
BAX	50599-2-lg	Proteintech, Wuhan, China		1:1000
BCL2	12789-1-AP	Proteintech, Wuhan, China		1:500
FSHβ	AB180489	Abcam, Cambridge, UK	1:100	1:1000
LHβ	AB150416	Abcam, Cambridge, UK		1:1000
LHβ	DF7140	AFFinity, Sterling, VA, USA	1:100	
β-actin	bs-0061R	Proteintech, Wuhan, China		1:1000
Goat anti-mouse lgG	20536-1-AP	Proteintech, Wuhan, China		1:2000
Goat anti-Rabbit lgG	SA00001-2	Proteintech, Wuhan, China	1:1000	1:2000

## Data Availability

The data that support the findings of this study are available in the methods and/or Supplementary Material of this article.

## Supplementary Materials

The following supporting information can be downloaded at: https://www.mdpi.com/article/10.3390/cells11060985/s1, Figure S1: The expression level of SMAD2 in different months of Hu sheep pituitary gland.

## Abbreviations

lncRNAs, long noncoding RNAs; HPO, hypothalamic–pituitary–ovarian; HPG, hypothalamic–pituitary–gonadal; ceRNAs, competing endogenous RNAs; GnRH, gonadotropin-releasing hormone; GnRHR, gonadotropin releasing hormone receptor; DAPI, 4′, 6-diamidino-2-phenylindole; BSA, bovine serum albumin; SMAD2, SMAD family member 2; BAX, BCL2 associated X; BCL2, BCL2 apoptosis regulator; FSHβ, follicle stimulating hormone subunit beta; LHβ, luteinizing hormone beta polypeptide; PCNA, proliferating cell nuclear antigen; TGF-β, transforming growth factor beta-1; TGFR1, TGF receptor-1 precursor.
