# Supplementary figures and images for: The Novel Competing Endogenous Long Noncoding RNA SM2 Regulates Gonadotropin Secretion in the Hu Sheep Anterior Pituitary by Targeting the Oar-miR-16b/TGF-β/SMAD2 Signaling Pathway
Source: Cells. 2022 Mar 14;11(6):985. doi: 10.3390/cells11060985 (PMC8947352; doi:10.3390/cells11060985)

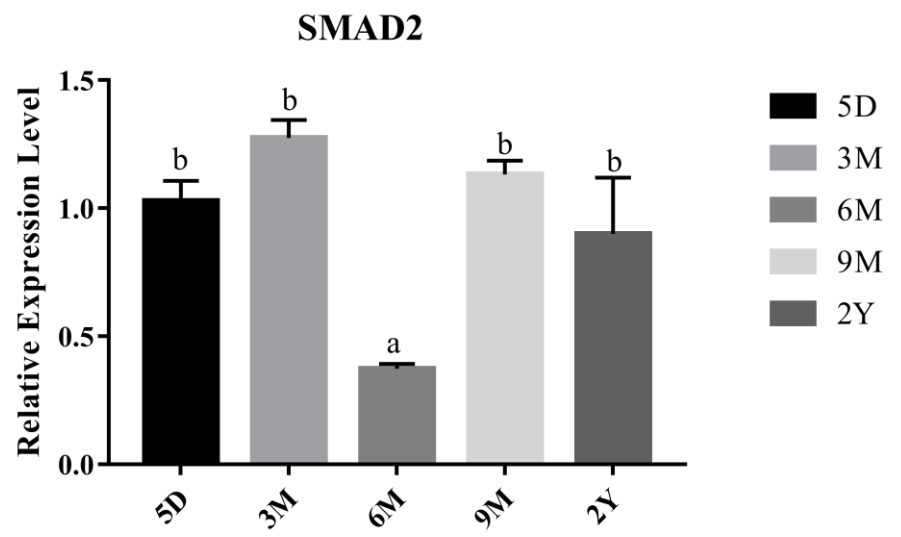

**Figure S1.** The expression level of SMAD2 in different months of Hu sheep pituitary gland.

Supplement: Supplementary file 1 [file cells-11-00985-s001.zip › cells-1592310-supplementary.pdf]
